# Cloning, structural modelling and characterization of VesT2s, a wasp venom hyaluronidase (HAase) from *Vespa tropica*

**DOI:** 10.1186/s40409-016-0084-5

**Published:** 2016-10-22

**Authors:** Prapenpuksiri Rungsa, Paroonkorn Incamnoi, Sophida Sukprasert, Nunthawun Uawonggul, Sompong Klaynongsruang, Jureerut Daduang, Rina Patramanon, Sittiruk Roytrakul, Sakda Daduang

**Affiliations:** 1 Protein and Proteomics Research Center for Commercial and Industrial Purposes (ProCCI), Department of Biochemistry, Faculty of Science, Khon Kaen University, Khon Kaen, 40002 Thailand; 2 Department of Chemistry, Faculty of Engineering, Rajamangala University of Technology Isan, Khon Kaen Campus, Khon Kaen, Thailand; 3 Chulabhorn International College of Medicine, Thammasat University (Rangsit Campus), Pathumthani, Thailand; 4 Division of Chemistry, Faculty of Science, Nakhon Phanom University, Nakhon Phanom, Thailand; 5 Department of Clinical Chemistry, Faculty of Associated Medical Sciences, Khon Kaen University, Khon Kaen, Thailand; 6 Genome Institute, National Center for Genetic Engineering and Biotechnology, National Science and Technology Development Agency (NSTDA), Pathumthani, Thailand; 7 Division of Pharmacognosy and Toxicology, Faculty of Pharmaceutical Sciences, Khon Kaen University, Khon Kaen, Thailand

**Keywords:** Wasp venom, *Vespa tropica*, Hyaluronidase (HAase)

## Abstract

**Background:**

Wasp venom is a complex mixture containing proteins, enzymes and small molecules, including some of the most dangerous allergens. The greater banded wasp (*Vespa tropica*) is well-known for its lethal venom, whose one of the major components is a hyaluronidase (HAase). It is believed that the high protein proportion and activity of this enzyme is responsible for the venom potency.

**Methods:**

In the present study, cDNA cloning, sequencing and 3D-structure of *Vespa tropica* venom HAase were described. Anti-native HAase antibody was used for neutralization assay.

**Results:**

Two isoforms, VesT2a and VesT2b, were classified as members of the glycosidase hydrolase 56 family with high similarity (42–97 %) to the allergen venom HAase. VesT2a gene contained 1486 nucleotide residues encoding 357 amino acids whereas the VesT2b isoform consisted of 1411 residues encoding 356 amino acids. The mature VesT2a and VesT2b are similar in mass and pI after prediction. They are 39119.73 Da/pI 8.91 and 39571.5 Da/pI 9.38, respectively. Two catalytic residues in VesT2a, Asp107 and Glu109 were substituted in VesT2b by Asn, thus impeding enzymatic activity. The 3D-structure of the VesT2s isoform consisted of a central core (α/β)_7_ barrel and two disulfide bridges. The five putative glycosylation sites (Asn79, Asn99, Asn127, Asn187 and Asn325) of VesT2a and the three glycosylation sites (Asn1, Asn66 and Asn81) in VesT2b were predicted. An allergenic property significantly depends on the number of putative *N*-glycosylation sites. The anti-native HAase serum specifically recognized to venom HAase was able to neutralize toxicity of *V. tropica* venom. The ratio of venom antiserum was 1:12.

**Conclusions:**

The wasp venom allergy is known to cause life-threatening and fatal IgE-mediated anaphylactic reactions in allergic individuals. Structural analysis was a helpful tool for prediction of allergenic properties including their cross reactivity among the vespid HAase.

## Background

Vespidae venom consists of complex mixtures of enzymes, proteins, peptides and small molecules responsible for many of the non-allergic and mild allergic reactions – such as local pain, inflammation and itching – as well as moderate and serious allergic reactions – such as anaphylaxis, and delayed hypersensitivity – including systemic toxic reactions, coagulopathy, acute renal failure and hepatotoxicity [1, 2]. Wasp venom contains many biological active compounds [3, 4]. The major allergens are phospholipase A1, hyaluronidase (HAase) and antigen 5 [5–8].

Venom HAase is an enzyme that hydrolyses hyaluronic acid (HA), one of the primary components of the extracellular matrix of vertebrates, which facilitates venom toxin diffusion into the tissue and blood circulation of the prey [9, 10]. HAase mainly acts as a “spreading factor” to enhance venom action. It has been identified in the venom of animals including snakes, bees, scorpions, fish, spiders, ants, wasps, caterpillars etc. [11–16]. Clinical studies have demonstrated that HAase is an “allergic factor” due to its ability to initiate pathogenic reactions in the majority of venom allergic patients [17–19]. It is also able to induce several anaphylactic IgE-mediated reactions in humans and has been suggested to be involved in the difficulties in the clinical diagnosis of venom allergic individuals [20–22]. The wasp venom HAase belongs to the hyaluronate glycanohydrolase family (EC 3.2.1.35), which degrades hyaluronic acid (HA) [23, 24]. Wasp venom HAase is responsible for the cross-reactivity of wasp and bee venom sera in patients as well [2, 25].

The greater banded wasp (*Vespa tropica*) is mostly distributed in the forest throughout Indochina peninsula including Thailand. It has a body length of up to 5 cm and its nest is usually found underground [26]. *V. tropica* is among the most venomous known insects. The lethal dose of its pure venom in experimental animals (LD_50_ of approximately 2.8 mg/kg in mice) is more potent than that of *V. affinis* venom [26, 27]. The potency of *V. tropica* venom has been reported to nearly stop the end plate potentials of *Drosophila* larvae in nerve-muscle preparation in response to treatment with this venom [28]. HAase was reported to be a major protein in *V. tropica* venom, where it is found by 2.5-fold the proportion observed in *V. affinis* venom [26]. The understanding of HAase in terms of biochemical and structural characterization of these wasps is important for the development of new tools for treating multiple stings and for diagnosis and therapy of allergic reactions caused by this venom. Therefore, the present study aimed to characterize HAase isoforms in the venom of *V. tropica* by analyzing its sequence and 3D modelling.

## Methods

### Animals

The wasps were collected from Siang Sao Village, Sri Songkram district, Nakorn Panom Province, northeastern Thailand [26]. The worker wasps were immediately shocked on ice. The venom reservoirs were removed from the sting apparatus by removing them from the bodies with forceps and squeezing. The droplets of venom and specimens of *V. tropica* were collected in a 1.5-mL microcentrifuge tube and then keep at −80 °C until use.

### RT-PCR and rapid amplification of cDNA ends (5′ and 3′ RACE)

Total RNA was extracted from the venom gland of *V. tropica* with TRIzol® reagent (Invitrogen, Life technologies, USA). RT-PCR was performed using the RevertAid First strand cDNA synthesis kit (Thermo Scientific, USA) as described in the instruction manual. PCR primers for the amplification of VesT2 were designed based on the sequence similarity of the conserved region of HAase from vespid venom and conserved nucleotide sequences corresponding to peptide sequences obtained from LC-MS/MS analysis (Table 1) [26]. The PCR was performed using green master mix reagent kits with Taq DNA polymerase (Promega, Singapore). The reaction contained 2 μg of cDNA, 1 UTaq DNA polymerase, 2.0 mM dNTP, 2.0 mM MgCl_2_ and 2 μM of primerin to a final volume of 25 μL under the following conditions: initial denaturation for 5 min at 94 °C, followed by 35 cycles at 94 °C (30 s); 55 °C (30 s); 72 °C (1 min) and a final extension at 72 °C for 5 min. The rapid amplification of cDNA ends (RACE) was performed with the RACE system (Invitrogen, Life Technologies, USA). The RACE PCR products were cloned into the pGEM®-T easy vector (Promega, USA) for sequencing [29].

**Table 1:** Primer design of gene-specific primers and PCR product size

Forward primer	Reverse primer	Product size (bp)
Full nucleotide sequence active form		
F4 GCCAGACTTTTCATGGAGGA (GSP1 for active)	R3 (7) ATCAGGGGTCAGTTCACGTC (GSP1 for active)	225
Adaptor primer (AP) 5′GGCCACGCGTCGACTAGTAC (T) 16 (GSP for cDNA synthesis of 3′ RACE system)	R4 (8) CGTCGGTCTCGGTAAGAAAA	
Abridged universal amplification primer (AUAP)	R5 (9) GTTCTCGTGCATCGCTGTAA	
VesT2a (F) *Nco*I **CCATGG**CTTCCGAGAGACC	VesT2a (R) *Xho*I **CTCGAG**TTAGTTAACGGCTTCTG	
Full nucleotide sequence inactive form		
F1 CGAAAAGGAAGCGTCGAATA (GSP for RT-PCR inactive form)	R1 CATCTTGTCGTTCTCGCTCA (GSP for RT-PCR inactive form)	190
F2 CTTCGGCGTCTATTTCAAGG (GSP for RT-PCR inactive form)	R2CCGCTAAGACAGTGGGGATA (GSP for inactive form)	229
Adaptor primer (AP) 5′GGCCACGCGTCGACTAGTAC (T) 16 (GSP for cDNA synthesis of 3′ RACE system)	R2 (1) CATCTTGTCGTTCTCGCTCA (GSP for RT-PCR inactive form)	
Abridged universal amplification primer (AUAP)	R1 (2) CCGCTAAGACAGTGGGGATA (GSP for inactive form)	

The bold letters represent the restriction sites

### Sequence analysis and structure modelling

The basic characterizations of the gene and protein sequences were analyzed using NCBI (http://www.ncbi.nlm.nih.gov/Database/index.html) and the basic local alignment search tool BLAST (http://www.ncbi.nlm.nih.gov/BLAST/). The phylogenic tree was created using CLUSTAL-X software analysis using the Neighbour-Joining method [30]. The three-dimensional models were created using the SWISS-MODEL program, the automated protein homology modelling template at ExPASY (Switzerland) and a template search with the Alignment Mode program from the protein database (http://swissmodel.expasy.org/) [31, 32]. The model was elucidated as a PDB file, and the structure was previewed and analyzed using Swiss-Pdb Viewer Deep View v4 software (http://www.expasy.org/). The molecular mass and isoelectric points were computed using the Compute pI/MW tool of ExPASy Bioinformatics (http://web.expasy.org/compute_pi/). The N-glycosylation sites were predicted using the CBS prediction severs (http://www.cbs.dtu.dk/services/NetNGlyc/) and compared with other wasp and bee venom HAases.

### Zymographic HAase activity assay

The *V. tropica* venom HAase activity was detected using 10 % SDS-PAGE containing hyaluronic acid as a substrate. Proteins were separated at 15 mA. The gel was incubated in 3 % Triton X-100 for 1 h with agitation in order to remove SDS and then transferred to the HAase assay buffer (0.15 M NaCl in 0.1 M formate buffer), rinsed twice with assay buffer, and then incubated on a rotating shaker for 16 h at 37 °C. The gels were rinsed twice with distilled water and stained in 0.5 % Alcian blue solution for 1 h. The destain was performed with 7 % acetic acid that was changed every 1 h until clear bands appeared on a pale blue background [33].

### Turbidity HAase activity assay

The turbidity HAase method followed the one by Pukrittayakamee et al. [34] with slight modifications. We mixed 0.5 mg/mL HA and buffer containing 0.15 M NaCl to a final volume of 100 μL and incubated for 30 min at 37 °C. The reaction was stopped using 200 μL of 2 % CTAB containing 2.5 % NaOH. The absorbance was measured at 405 nm. The turbidity reducing activity was expressed as the percentage of remaining HA by taking the absorbance of the tube at 100 % in which no enzyme was added. The optimal pH of the venom HAase was determined by changing the buffers of the enzymatic turbidimetric venom HAase activity assay as follows: 0.2 M formate buffer, pH 2–4; 0.2 M acetate buffer, pH 5–6; 0.2 M Tris–HCl buffer, pH 7–10.

### Mouse anti-hyaluronidase serum

The HAase band from zymographic gel were cut and frozen at −70 °C overnight, the gel was freeze-dried and ground. Anesthetized mice were subcutaneously immunized with gel swollen in PBS buffer (135 mM NaCl, 1.5 mM KH_2_PO_4_, 2.5 mM KCl, and 8 mM Na_2_HPO_4_) emulsified with Freud’s complete adjuvant. Mice were four times boosted with the antigen emulsified with incomplete Freund’s adjuvant. After retro-orbital plexus bleeding, blood was kept at 4 °C for 12 h and centrifuged at 10000 × *g* for antiserum collection.

### Western immunoblotting

Proteins were separated by SDS-PAGE and blotted onto a nitrocellulose membrane (Bio-Rad, USA). After being eletrotransferred, the membrane was incubated with 5 % nonfat dry milk for 1 h, anti-HAase antibody for 1 h and goat anti-mouse IgG linked alkaline phosphatase (1:500) for 1 h. The blotted bands were detected by a substrate kit (GE Healthcare, Sweden). The membrane was intensive washed before the next incubation.

### Neutralization assay

Crickets (*Gryllus* sp.) were abdominally injected with venom pre-incubated with anti-HAase serum 10 min before considered paralyzed. The paralyzed crickets were defined as those that could return from the overturned position.

## Results

### Sequence and structural modelling analysis of VesT2s

**Fig. 1:**
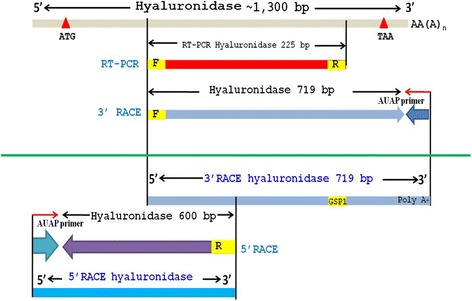
The cloning strategies of *Vespa tropica* HAase (VesT2s). The 357 amino acid sequence of VesT2s was deduced by overlapping of the VesT2s HAase gene and determined by using RT-PCR and RACE-PCR

The completed cDNA sequence was designed according to the peptide sequences obtained by LC-MS/MS and the sequence similarities of the conserved region of the other wasp venom hyaluronidases [26]. The primers were designed from nucleotide sequences based on the conserved region corresponding to the peptide sequence. The nucleotide fragment was obtained via RT-PCR. The 3′ and 5′ end were determined using RACE. They were completely overlapped (Fig. 1). Two HAase isoforms, VesT2a and VesT2b, were obtained.

**Fig. 2:**
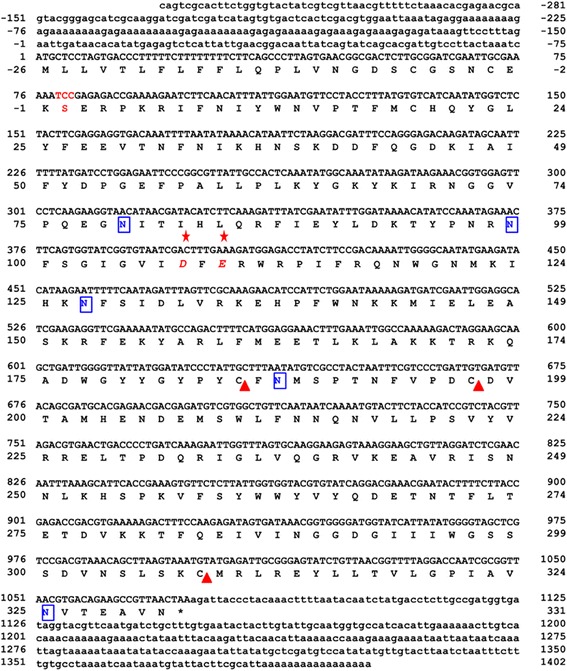
The completed nucleotide sequence and deduced amino acid sequence of *Vespa tropica* HAase a (VesT2a). The numbering corresponded to the VesT2a sequence. The 5′ and 3′ UTRs were indicated by the small letters. The red stars (✩) (Glu and Asp) indicate active sites of HAase whose activity is highlighted by the red letters. The stop codon was indicated with an asterisk (*). Disulfide bonds were labelled with triangle marks (△): (C_19_-C_308_ and C_185_-C_197_). Five potential immunogenic sites were indicated with blue boxes (▯)

The full length VesT2a cDNA was 1,683 bp in length and contained 281 bp of the 5′-untranslated region (UTR) and 328 bp of the 3′-UTR; 1,074 bp of an open reading frame (ORF) encoded a protein of 357 amino acids (Fig. 2). The primary sequence of the deduced VesT2a contained 357 amino acid residues including a predicted signal peptide (26 amino acid residues) that was rich in the amino acids Asn, Lys, Ile and Leu, with a predicted mature pI and molecular mass of 8.91 and 39,119.73 Da, respectively. The five potentially immunogenic *N*-glycosylated sites (Asn-Xaa-Thr/Ser, where Xaa is any amino acid residue except proline) on residues Asn79, Asn99, Asn127, Asn187 and Asn325 were predicted. The two disulfide bridges (Cys19-Cys308 and Cys185-Cys197) were responsible for the stabilization of protein structure (Fig. 2).

**Fig. 3:**
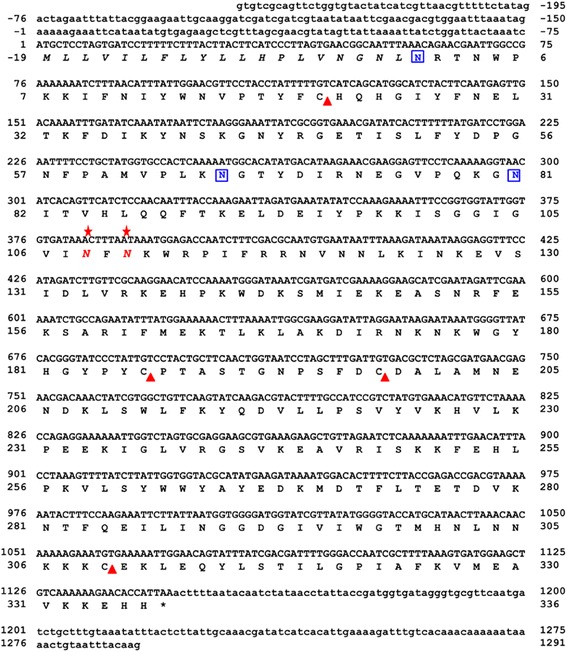
The completed nucleotide sequence and deduced amino acid sequence of *Vespa tropica* HAase b (VesT2b). The numbering corresponded to the VesT2b sequence. The 5′ and 3′ UTRs were indicated by the small letters. Disulfide bonds were labelled with triangle marks (△): (C_21_-C_310_ and C_187_-C_199_). Three potential immunogenic sites were indicated with blue boxes (▯)

Additionally, a putative HAase isoform was recently suggested as another component appearing in the 2D-PAGE profile from the corresponding cDNA of VesT2b [26]. It had an experimental mass of approximately 46 to 47 kDa. After amplification using several strategies (Fig. 1), the VesT2b precursor contained a 195-bp 5′-UTR, a 145-bp 3′-UTR and an 1146-bp ORF. The ORF consisted of a 57-bp predicted signal sequence, which corresponded to 19 amino acid residues, and a 1089-bp mature sequence encoding 337 amino acids. The primary sequence of the deduced VesT2b mature protein contained 337 amino acid residues (996 bp) and was rich in the amino acids Lys, Asn and Ile with a theoretical pI 9.38 and a predicted molecular mass of 39571.5 Da. The three potentially immunogenic *N*-glycosylated sites (Asn1, Asn66 and Asn81) and the two disulfide bridges (Cys21-Cys310 and Cys187-Cys199) were predicted (Fig. 3). The VesT2s mature amino acid sequence in these studies had 61.52 % homology; the two catalytic residues in VesT2a, Asp107 and Glu109, were substituted byAsn in VesT2b (Fig. 3).

**Fig. 4:**
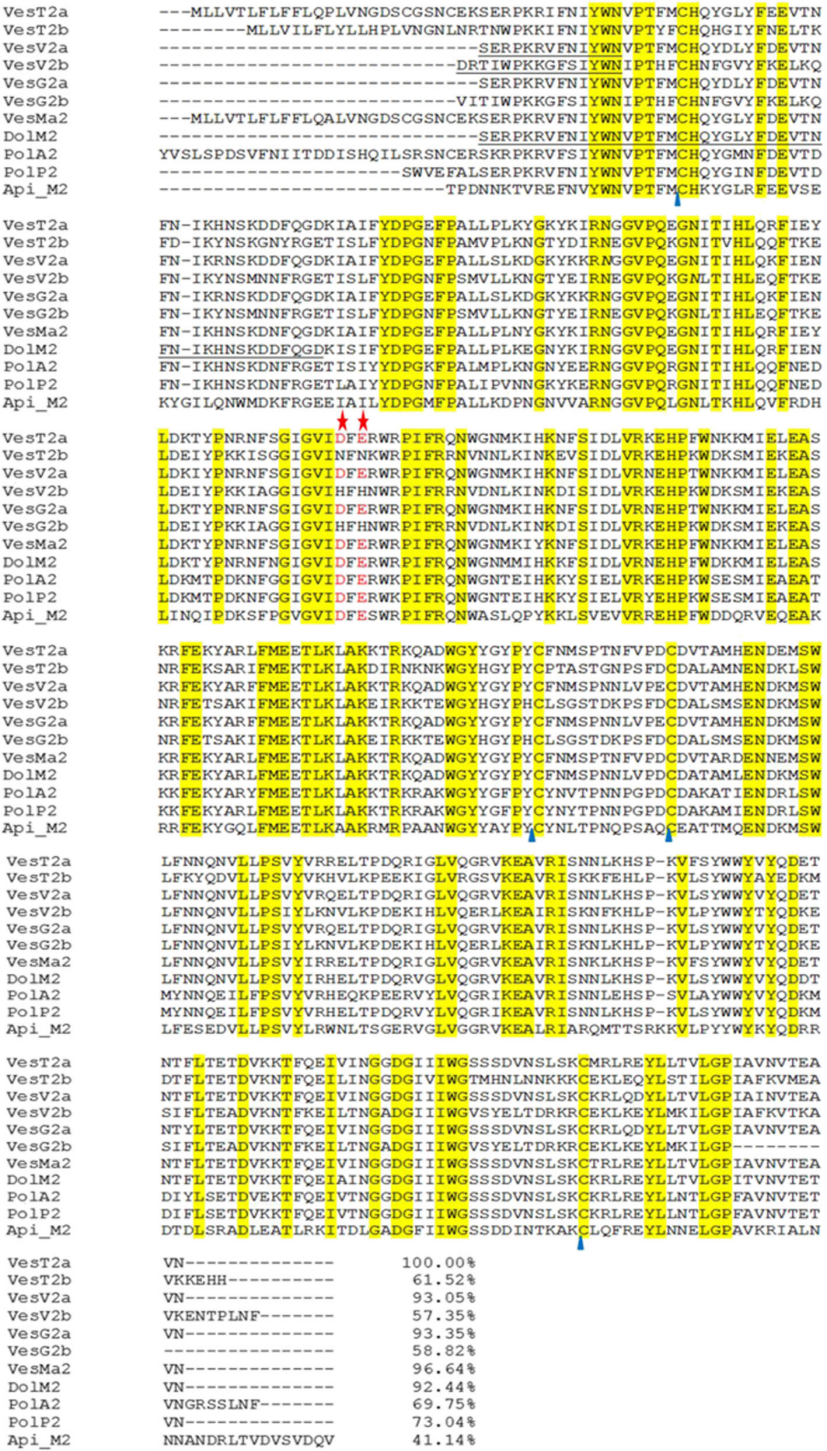
Sequence alignment of the deduced amino acid sequence of *Vespa tropica* HAase with other allergen venom HAases. VesT2s was aligned with the known HAases; VesV2a and VesV2b (*Vespula vulgaris*; active and inactive forms), VesG2a and VesG2b (*Vespula germanica*; active and inactive forms), *Vespa magnifica* (VesMa2), DolM2 (*Dolichovespula maculata*), PolA2 (*Polistes annularis*), PolP2 (*Polybia paulista*) and Api_M2 (*Apis mellifera*). The shaded yellow alignment corresponds to conserved residues in HAase. The N-terminus was shown in the underlined amino acid sequence obtained by Edman sequencing. The catalytic residues (D and E letters) are indicated with the red stars (✩). The conserved cysteine positions among the HAase are indicated with blue triangles (△)

**Fig. 5:**
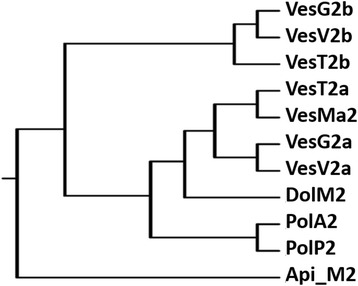
The phylogenic tree of HAases from insect venoms

**Fig. 6:**
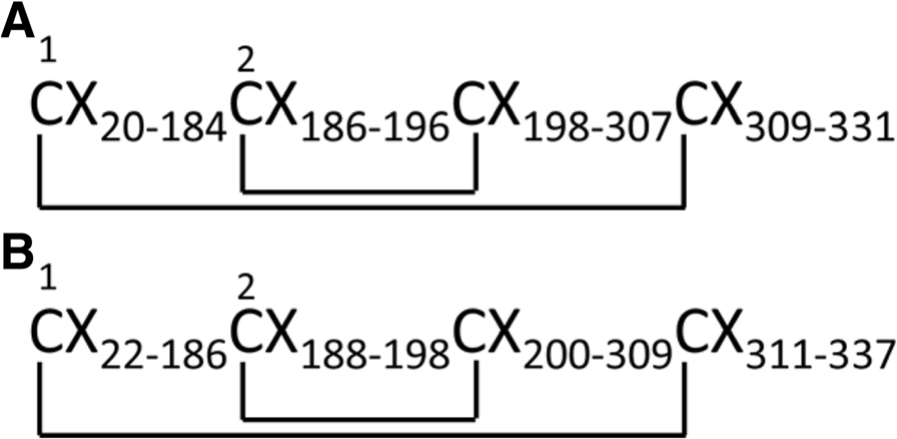
The disulfide linkages of VesT2s [(**a**); VesT2a and (**b**); VesT2b]. The two disulfide bridges (VesT2a; C_19_-C_308_ and C_185_-C_197_, VesT2b; C_21_-C_310_ and C_187_-C_199_) are linked via a solid line

The multi-sequence alignment of venom HAases (Fig. 4) showed the highest BLAST homology score (>90 % identity) of VesT2a to many HAases, VesV2a of *Vespula vulgaris*, VesG2a of *Vespa germanica*, VesMa2 of *Vespa magnifica*, and Dol m 2 of *Dolichovespula maculata*, suggesting high evolutionary conservation among these species. The catalytic residues (Asp107 and Glu109) were conserved in active venom VesT2a [20, 22, 35, 36]. The phylogenetic tree analysis revealed the highest similarity of VesT2a to VesMa2, which was higher than that of the VesT2b depicted in the phylogenetic tree of the insect HAase (Fig. 5). VesT2s contained cysteine residues that were conserved among venom HAases and also formed two disulfide bonds (Fig. 6a and b). For VesT2a, these were Cys19-Cys308 and Cys185-Cys197, whereas they were Cys21-Cys310 and Cys187-Cys199 in the structure of VesT2b.

**Fig. 7:**
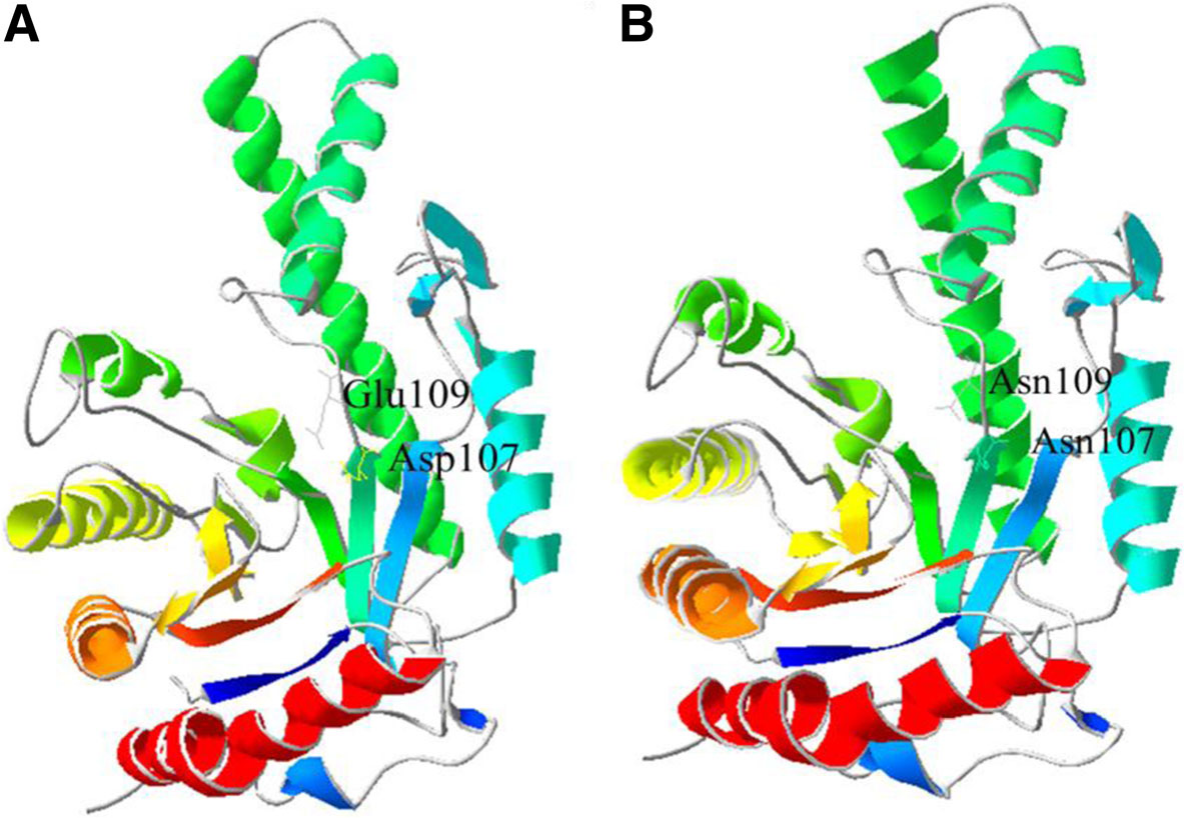
The predicted three-dimensional structural modelling of VesT2s. The *Vespa tropica* HAase [VesT2a (**a**) and VesT2b (**b**)] modelling used VesV2 as a template (*Vespula vulgaris*, PDB accession number 2ATM_A). VesT2s was generated with SWISS-MODEL automated software and was visualized by the Swiss-Pdb Viewer Deep View v4.0 program. The two catalytic sites of VesT2a (Glu109 and Asp 107) were changed to Asn in VesT2b

VesV2 (PDB ID: 2ATM) was used as a template for computational homology modelling. VesT2a and VesT2b showed 92.28 % and 62.69 % sequence identity to VesV2, respectively, with an E value of 6.19e^−153^. Based on the model, VesT2a and VesT2b displayed a central core (α/β)_7_ consisting of seven helices and seven beta-sheets belonging to family 56 of glycoside hydrolases [37] (Fig. 7).

### HAase activity of wasp venom VesT2a

**Fig. 8:**
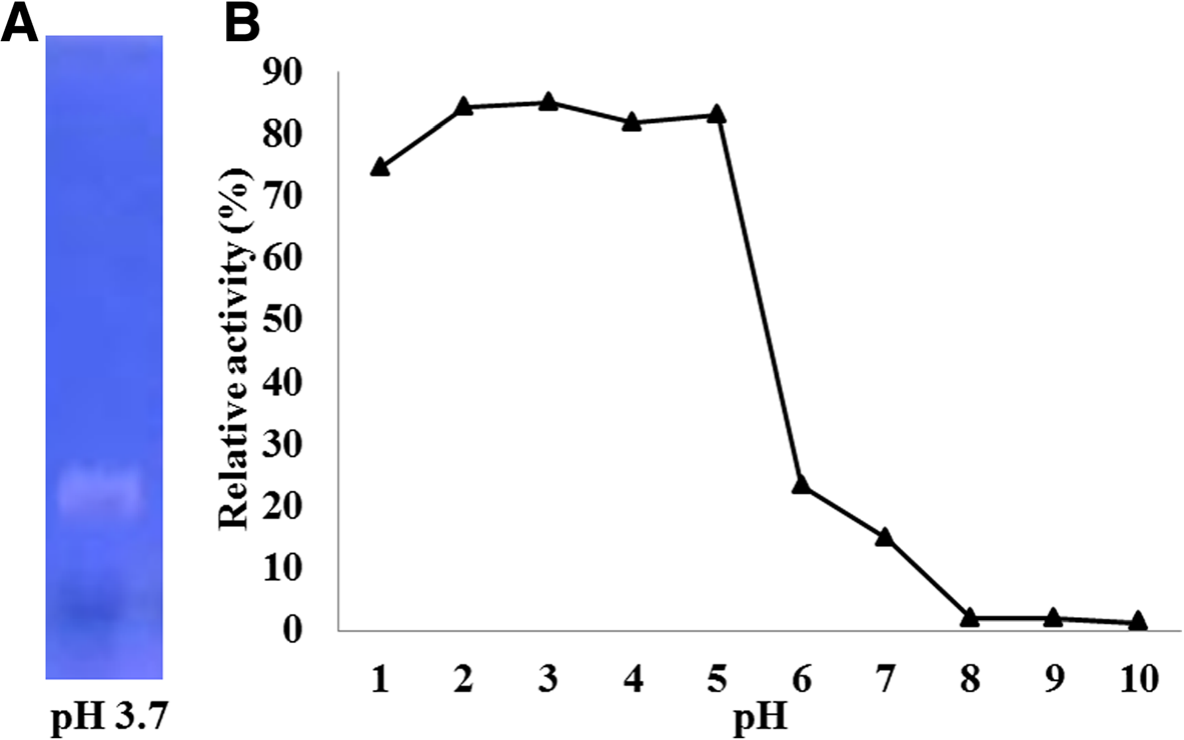
The HAase activity. VesT2a: *V. tropica* venom was tested for specific HAase activity by the (**a**) zymographic method and (**b**) optimal pH using turbidimetric assay. For the zymogram HAase activity assay, samples were analysed using 10 % SDS-PAGE containing hyaluronic acid under reducing conditions. The zymgraphic gel was developed overnight at 37 °C under pH 3.7. For the turbidimetric assay, the enzymatic activity of VesT2a, *V. tropica* venom was tested at various pHs using hyaluronic acid as a substrate at 37 °C

*V. tropica* venom VesT2a was tested for specific HAase activity using zymographic method at 37 °C, pH 3.7, under reducing conditions. The result showed a transparent band (Fig. 8a). The turbidity method was used to determine the optimal pH of venom HAase, with hyaluronic acid as substrate. The *V. tropica* HAase, VesT2a, had an optimal pH of about 3 (Fig. 8b). It clearly displayed a higher HAase activity between pH 2 and 5.

### Neutralization assay

**Fig. 9:**
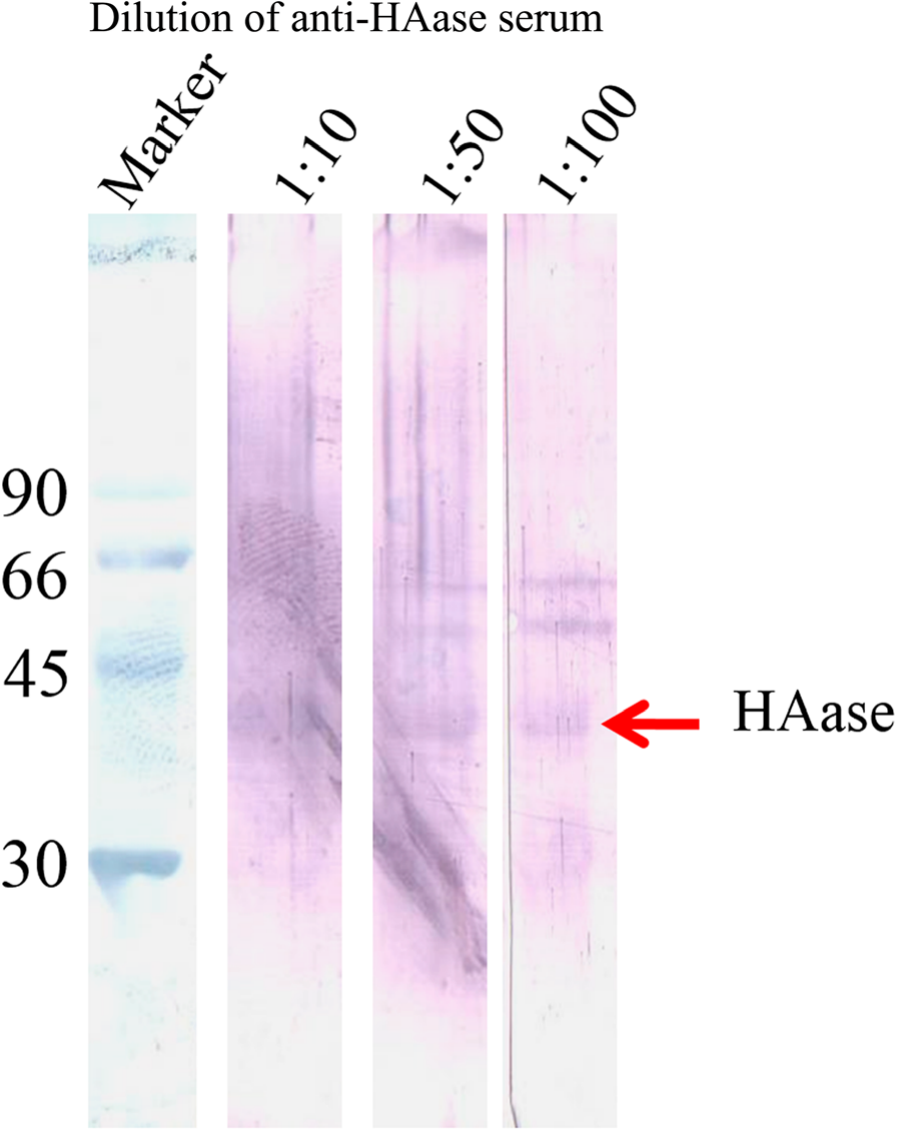
Western immunoblotting analysis of venom HAase with an anti-HAase serum. Lane 1: molecular weight marker. Lanes 2–4: HAase was incubated with different dilutions of anti-HAase serum. Venom HAase is indicated by the arrow

The Western immunoblotting revealed the specificity of antibodies to their antigens when the titer was 1:100 (Fig. 9). The anti-HAase serum was able to reduce venom toxicity (Table 2). Non-paralyzed crickets were observed at the ratio 1:12 (venom: antiserum).

**Table 2:** The neutralization assay of *V. tropica* venom against anti-HAase serum in crickets (*Gryllus* sp.)

*V. tropica* venom: Anti-HAase serum (μL/μL)	Neutralized crickets/total crickets after injections with *V. tropica* venom and anti-HAase serum
1:4	2/4
1:8	1/4
1:12	0/4

## Discussion

In this study, we described the identification, biochemistry, bioactivity and structural characteristics of the HAase from the venom of greater banded wasp *V. tropica*. This study describes the existence of two isoforms of VesT2s, VesT2a and VesT2b. The primary sequence of VesT2a and VesT2b were clearly isoenzymes with 61.52 % similarity but with different molecular masses and pIs of the mature sequence (357 amino acids/39119.73 Da/pI 8.91 and 337 amino acids/39571.53 Da/pI 9.38, respectively). Mass differences were mainly estimated from amino acid variations, including the degree of glycosylation of VesT2s. However, they were classified into the same family of glycoside hydrolase family 56 by sequence similarity. This phenomenon also occurs with HAases in many species, such asVesV2a and VesV2b, the HAase isoenzymes in *Vespula vugaris* venom. VesV2a and b share 58 % amino acid identity to each other [5, 20].

Rungsa et al. [26] indicated that the mass of HAase in *V. tropica* venom was approximately 43 kDa after analysis by denaturing two-dimensional electrophoresis, which was confirmed by peptide mass fingerprinting. However, the mature sequence of HAase in this study, VesT2s, was smaller in size, with approximately 39 kDa. The molecular mass of about 43 kDa of native VesT2s was not surprising, since wasp venom HAase is a glycoprotein whose differences in estimated values of theoretical pI and molecular masses are frequent [9, 38, 39].

The phylogenetic tree demonstrated that VesT2a is found in the same cluster of active HAase from insect venoms. VesT2b is also found in a cluster of inactive HAase from insect venoms [2, 20, 35, 38, 40]. The enzyme function of VesT2s is different because of two catalytic residues in VesT2a, Asp107 and Glu109. Both are substituted by Asn in VesT2b that has no HAase enzymatic activity towards various substrates [20, 35, 41]. The less acidic Asn cannot act as a proton donor as the acidic amino acids, Asp and Glu [36, 37].

Glycosylation sites are the most common post-translational modification of many insect venom proteins as they contribute to biological activity, immunogenicity, and solubility, stability and protease resistance. VesT2s represents one of the strongest conserved hymenoptera venom allergens in wasps, yellow jackets and honeybees [42, 43]. VesT2a is highly similar to VesMa2 (*Vespa magnifica* HAase) while VesT2b is close to VesV2b (*Vespula vugaris* HAase b). *V. vugaris* and *V. magnifica* also belong to the Vespidae family [20, 35, 40]. Therefore, we presume that the VesT2s isoform might have a similar structure and allergic properties.

Insect venom allergies are known to cause life-threatening and sometimes fatal IgE-mediated anaphylactic reactions in allergic individuals. Approximately 30 to 50 % of patients with insect venom allergies have IgE antibodies that react with both honeybee and yellow jacket venom [44]. Previous studies have demonstrated that human IgE antibodies share cross reactive B-cell epitopes with various venom HAases to VesV2 [2, 25]. Honeybee and yellow jacket venom HAases with a molecular mass of approximately 42–45 kDa are considered to be major allergen proteins and are responsible for cross-reactivity with allergen patient sera [44]. The venom HAase in insects are classical allergens responsible for cross-reactivity. Nevertheless, the cross-reactivity of venom HAase was identified by cross reactive carbohydrate determinants (CCD) [42, 45]. Previous studies showed that VesV2s and VesMa2 were isoallergens that significantly differed in the number of putative *N*-glycosylation sites (Table 3) [9, 22, 37, 40]. According to the sequencing analysis of VesT2s, it contains five *N*-glycosylation sites in VesT2a (Asn79, Asn99, Asn127, Asn187 and Asn325) and three *N*-glycosylation sites in VesT2b (Asn1, Asn66 and Asn81). Based on this data, we speculated about a high degree of CCD. These data are potentially relevant, especially regarding to the cross-reaction [40, 46].

**Table 3:** *N*-glycosylation in wasp venom HAase. Asn-Xaa-Ser/Thr residues represent the possible *N*-glycosylation sites predicted by NetNGlyc 1.0 Server (N-glycosylation in *V. vulgaris* and *V. magnifica* HAase was obtained in the experiment in the native form)

*V. tropica* (this study)		*V. vulgaris* [22]		*V. magnifica* [9]
VesT2a (active HAase)	VesT2b (inactive HAase)	VesV2a (active HAase)	VesV2b (inactive HAase)	VesMa2 (active HAase)
Asn79	Asn1	Asn79	Asn66	Asn105
Asn99	Asn66	Asn99	Asn81	Asn125
Asn127	Asn81	Asn127		Asn153
Asn187				Asn351
Asn325				

Via the turbidity method, *V. tropica* venom HAase was clearly active at a pH ranging from 2 to 5 (more than 80 % of relative activity) with an optimal pH of approximately 3 to 4. At pH 6 to 10, the activity reduced and no detectable activity was observed within the range of basic pH (8–10). Therefore, VesT2a was predicted as a strong acid HAase. However, the optimal pH (3 to 4) in this study was quite different from those of other wasp venoms, such as *V. vulgaris* (pH 5–6), *V. germanica* (pH 5–6) and *D. maculata* (pH 5–6) [47]. Generally, the activity of HAases to degrade hyaluronic acid (HA) have an optimal pH ranging from 3 to 4, which is in accordance with VesT2a in this study (Table 4) [48].

**Table 4:** The biochemical and physiological characterization of vespid venom

Species	Molecular weight	Pi	Optimal pH	Reference
*D. maculate*	39	5-6	5–6	[47]
*V. germanica*	42	5–6	5–6	[47]
*V. vulgaris*	43	5–6	5–6	[47]
*V. tropica*	46–47	8.91	3–4	Current study
*P. paulista*	43.277	8.77	ND	[9]

A previous study showed the high potency of *V. tropica* venom (PD_50_ ~ 3 μg/g body weight of cricket) [26]. Venom HAase, a “spreading factor”, is well-known for its toxin-enhancing activity. Therefore, the anti-HAase serum was produced. The anti-HAase serum shows neutralizing efficiency against crude venom by ratio the ratio of 1:12 (venom:antiserum). Inhibition of HAase activity not only prevents local tissue damage, but also retards the venom toxin diffusion into the tissues and blood circulation, resulting in the delay of fatal outcomes in several cases [13]. HAase activity may play a vital role in allergenicity and toxicity of venoms.

## Conclusions

Hymenoptera venom showed cross-reactivity with bee and wasp venoms [2]. The allergic responses to wasp venom are known to cause life-threatening and fatal IgE-mediated anaphylactic reactions in sensitive individuals. The cross reactivity among the hyaluronidase from yellow jacket and bee venom are presumably induced by CCDs, but less often shared by peptide epitopes [19]. Knowledge on the structural determinants responsible for the allergic potency is expected to have important clinical implications.
